# Prepancreatic postduodenal portal vein: a case report and review of the literature

**DOI:** 10.1186/s13256-016-1165-3

**Published:** 2017-01-04

**Authors:** Naeem Goussous, Steven C. Cunningham

**Affiliations:** Department of Surgery, Pancreatic and Hepatobiliary Surgery Service, Saint Agnes Hospital, 900 Caton Avenue, MB 207, 21229 Baltimore, MD USA

**Keywords:** Portal vein, Anomaly, Pancreatic surgery, Case report, Literature review

## Abstract

**Background:**

Prepancreatic postduodenal portal vein is extremely rare, with only 13 cases reported in the literature.

**Case presentation:**

A 55-year-old white woman presented to our emergency department with abdominal pain. She underwent a computed tomography of her abdomen, which showed a portal vein coursing anterolaterally to her pancreas and posteriorly to the first portion of her duodenum, constituting a prepancreatic postduodenal portal vein. Imaging revealed choledocholithiasis, requiring endoscopic sphincterotomy, but due to a history of a gastric bypass procedure, she was lost to follow-up after being referred to an advanced endoscopist. This represents the 14th reported case of prepancreatic postduodenal portal vein.

**Conclusions:**

Awareness of this rare anomaly is paramount, and will help surgeons and interventional radiologists to avoid complications related to inadvertent injury to the portal vein, which could be life-threatening.

## Background

Anatomical variants of the portal vein (PV) are rare and one of the least common of these is the development of a prepancreatic postduodenal portal vein (PPPV), an anomaly in which the PV lies anterior to the pancreas and posterior to the duodenum instead of being posterior to both the pancreas and the duodenum. Awareness of this very rare anomaly is very important especially prior to any surgical or percutaneous interventions involving the biliary tree, liver, or pancreas, to avoid potentially devastating injuries to the PV which could result in liver ischemia or massive hemorrhage. We found only 13 cases reported in the literature, and add this 14th case [1–12].

## Case presentation

A 55-year-old white woman presented to our emergency department with acute onset of abdominal pain, which was associated with nausea and multiple bouts of emesis. Her past medical history included deep venous thrombosis currently treated with warfarin and a history of rheumatoid arthritis. She stopped smoking tobacco 10 years prior to presentation and she denied any significant alcohol intake. She had no relevant environmental exposures. Her past surgical history was significant for a Roux-en-Y gastric bypass (RYGB).

**Fig. 1:**
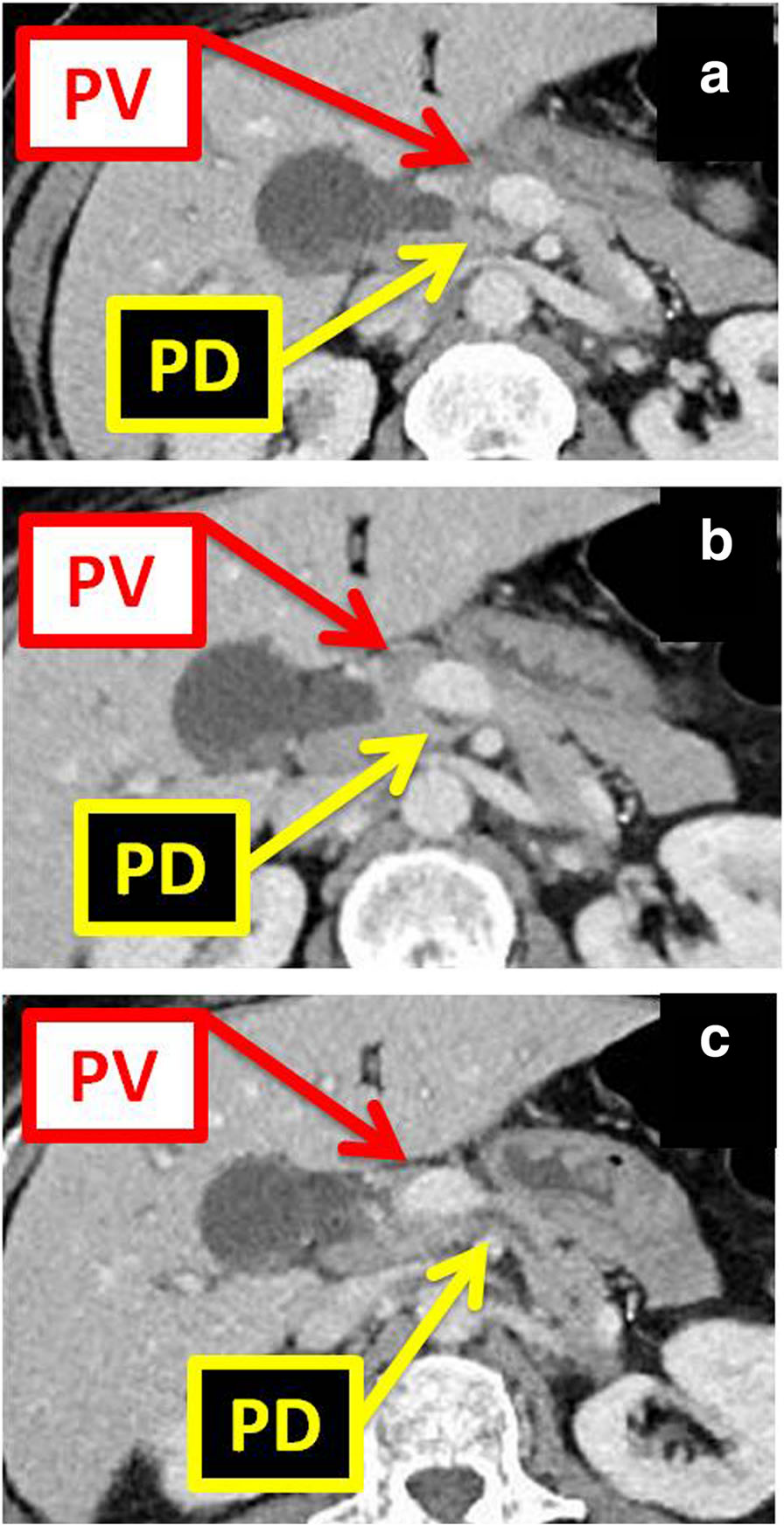
Axial images of computed tomography scan of the abdomen. The images show the portal vein in between the duodenum and the pancreatic duct. Three sequential cuts shown: **a**–**c** caudal to cephalad. *PD* pancreatic duct, *PV* portal vein

**Fig. 2:**
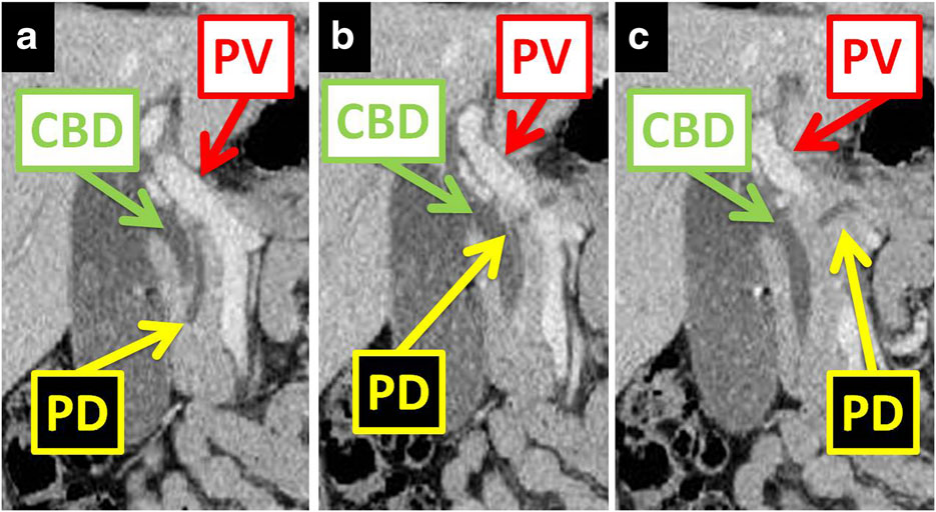
Coronal images of computed tomography of the abdomen. The images show linear configuration of the portal vein and common bile duct, with the pancreatic duct coursing posterior to the portal vein. Three sequential cuts shown: **a**–**c** anterior to posterior. *CBD* common bile duct, *PD* pancreatic duct, *PV* portal vein

**Fig. 3:**
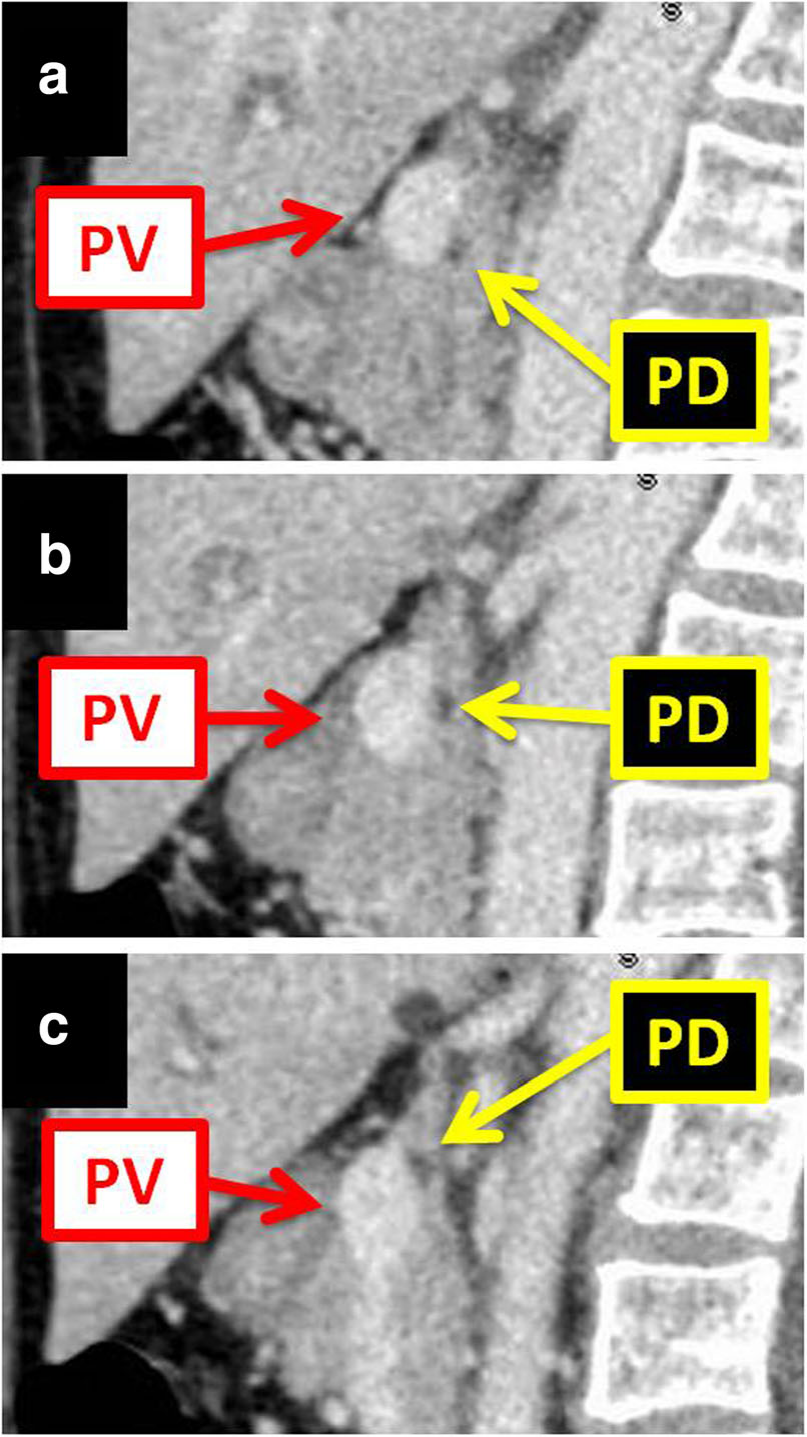
Sagittal images of computed tomography of the abdomen. The images show the pancreatic duct coursing posterior to the portal vein. Three sequential cuts shown: **a**–**c** right to left. *PD* pancreatic duct, *PV* portal vein

On examination her vital signs were normal. She was alert and oriented to person, place, and time. Her memory and speech were normal. Her strength was normal and symmetric. Her sclerae were anicteric. Her breathing was nonlabored and her breath sounds clear. An abdominal examination was unremarkable except for abdominal pain in her right upper quadrant, without evidence of guarding and a negative Murphy’s sign. A laboratory evaluation showed an elevated white blood cell count of 14.4 K/mm^3^ on admission and 7.62 K/mm^3^ on discharge. Aspartate transaminase, alanine transaminase, and alkaline phosphatase were 551 U/L, 256 U/L, and 144 U/L respectively, on admission, peaking at 2195 U/L, 1244 U/L, and 201 U/L 1 day later, and normalizing by discharge 3 days later: 101 U/L, 258 U/L, and 180 U/L. Her bilirubin was 0.9 mg/dL on admission and discharge, having peaked at 3.6 mg/dL 2 days after admission. Right-upper-quadrant ultrasound showed evidence of a gallbladder wall measuring 3.7 mm in thickness. She also underwent a computed tomography (CT) of her abdomen and pelvis, which showed her PV to be coursing anterolaterally to her pancreas and posteriorly to the first portion of her duodenum (Figs. 1, 2, and 3). Magnetic resonance cholangiopancreatography showed a filling defect in her common hepatic duct. Due to her altered upper gastrointestinal anatomy following RYGB, an attempt to perform endoscopic retrograde cholangiography (ERC) was unsuccessful. Instead she underwent placement of a percutaneous gastrostomy tube to her remnant stomach in preparation for a transabdominal antegrade ERC. Unfortunately, she subsequently left town and was lost to follow-up. She was, however, reached by phone and reported that an ERC was eventually performed, the duct was cleared, and the gastrostomy tube removed, but other details of her case were unknown and unobtainable.

## Discussion

PPPV is a rare anomaly first described in 1972 by Brook and Gardner. It was discovered incidentally during exploration for choledocholithiasis [12]. Only 13 more cases, including ours, have since been reported with most of the reports coming from Japanese groups (Table 1). It is most commonly found incidentally during diagnostic imaging (11/14) and the rest were found during exploration for unrelated pathology (3/14). This variation is much rarer as compared to other PV anomalies including preduodenal PV, which was first described by Knight in 1921 [13]. The existence of many diverse rare anomalies and variants, especially those with potential clinical ramifications such as PPPV, lends support to the argument for subspecialist radiologists [14], who are more likely to accurately describe these cases.

**Table 1:** Cases of prepancreatic postduodenal portal vein

Author	Year	Age and gender	Principle diagnosis	How it was discovered
Brook and Gardner [12]	1972	84 F	Choledocholithiasis	Laparotomy
Matsumoto *et al.* [6]	1983	64 M	Carcinoma of the bile duct	Laparotomy
Dumeige *et al.* [11]	1989	49 M	Chronic pancreatitis	Laparotomy
Matsui *et al.* [7]	1995	66 F	Carcinoma of the bile duct	PV
Yasui *et al.* [5]	1998	65 M	Cecal cancer	PV
Ozeki *et al.* [4]	1999	62 F	Liver metastasis from rectal cancer	CT scan
Tanaka *et al.* [3]	2000	61 M	Carcinoma of the bile duct	
Inoue *et al.* [10]	2003	50 M	Gastric cancer	CT scan
Jung *et al.* [2]	2005	28 F	Cholelithiasis	CT scan
Tomizawa *et al.* [8]	2010	74 M	Colorectal metastasis to the liver	CT scan
		74 F	Breast cancer	CT scan
Jain *et al.* [9]	2013	56 F	Autoimmune hepatitis	CT scan
Shimizu *et al.* [1]	2014	85 F	Carcinoma of ampulla of Vater	CT scan
Current case	2016	55 F	Choledocholithiasis	CT scan

*CT* computed tomography, *F* female, *M* male, *PV* portal venogram

**Fig. 4:**
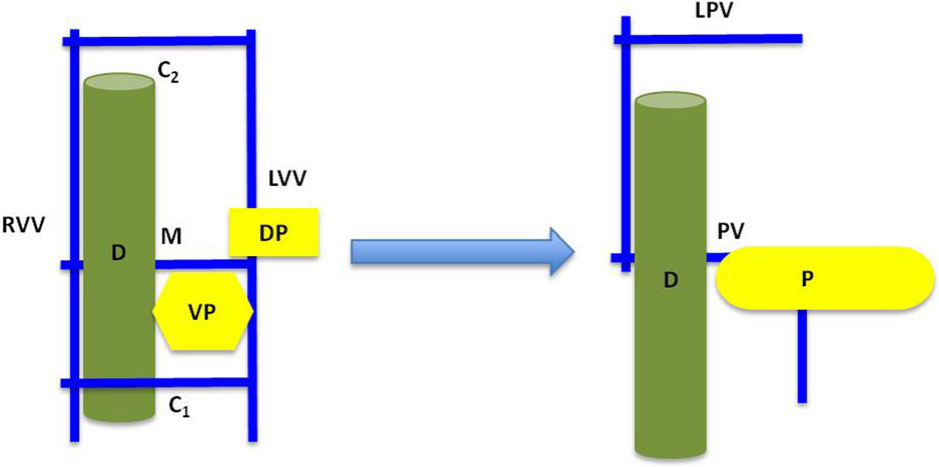
Normal development of the portal vein. The two vitelline veins are connected by three anastomoses (cranial, middle, and caudal). The dorsal pancreatic bud lies cephalad and ventral to the middle anastomosis. The duodenum lies ventral to the middle anastomosis and dorsal to the caudal anastomosis. During embryogenesis the caudal segment of the right vitelline vein and cranial segment of the left vitelline vein regress along with the caudal anastomosis. The pancreatic buds rotate in a clockwise fashion (viewed from caudal perspective) and fuse together resulting in a portal vein in the usual position posterior to the duodenum and the pancreas. *C1* caudal anastomosis, *C2* cranial anastomosis, *D* duodenum, *DP* dorsal pancreatic bud, *LPV* left portal vein, *LVV* left vitelline vein, *M* middle anastomosis, *P* pancreas, *PV* portal vein, *RVV* right vitelline vein, *VP* ventral pancreatic bud

The embryogenesis of the PV was described in 1969 by Marks [15]. During the fourth week of gestation, venous blood from the gut is drained by two vitelline veins (right and left), which are connected by three anastomoses (Fig. 4): one cranial, one middle, and one caudal. The cranial and caudal anastomoses are ventral to the duodenum, whereas the middle anastomosis is dorsal to the duodenum. During fetal development regression occurs of the caudal anastomosis, the inferior portion of the right vitelline vein, and the superior part of the left vitelline vein (Fig. 4). The middle anastomosis becomes the main PV and the cranial anastomosis becomes the left PV.

**Fig. 5:**
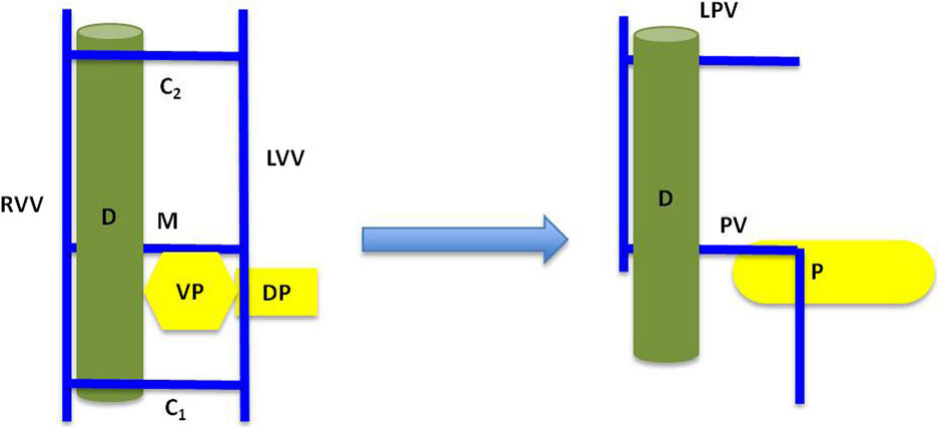
Development of the prepancreatic postduodenal portal vein. In this variant the dorsal pancreatic bud lies not cephalad and ventral, as is in normal development (Fig. 4), but rather the opposite: caudal and dorsal to the middle anastomosis and the caudal segment of the left vitelline vein, respectively. Rotation of the pancreatic buds in a clockwise fashion results in a portal vein posterior to the duodenum but anterior to the pancreas. *C1* caudal anastomosis, *C2* cranial anastomosis, *D* duodenum, *DP* dorsal pancreatic bud, *LPV* left portal vein, *LVV* left vitelline vein, *M* middle anastomosis, *P* pancreas, *PV* portal vein, *RVV* right vitelline vein, *VP* ventral pancreatic bud

**Fig. 6:**
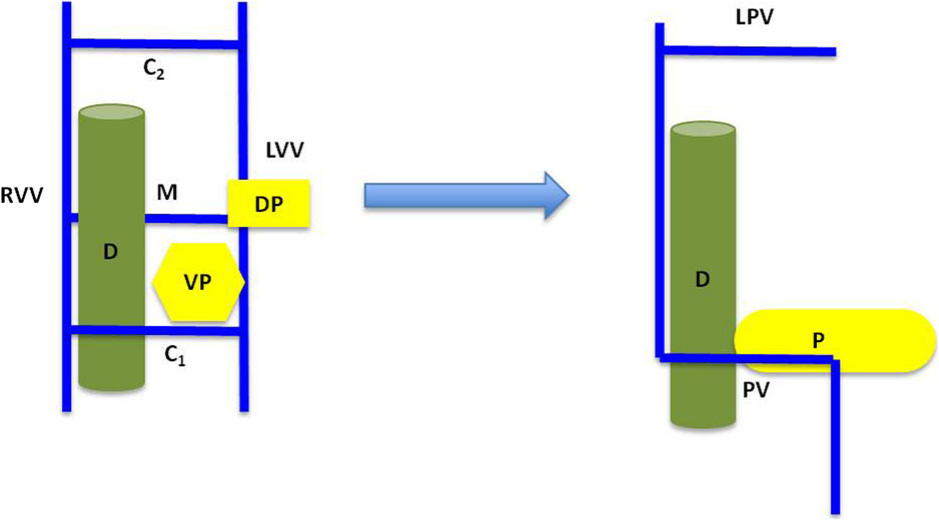
Development of the preduodenal portal vein. In the preduodenal variant, the middle anastomosis involutes and the caudal anastomosis forms the portal vein. The ventral pancreas rotates in a counter clockwise direction to fuse with the dorsal pancreatic bud. This results in a portal vein anterior to the duodenum. *C1* caudal anastomosis, *C2* cranial anastomosis, *D* duodenum, *DP* dorsal pancreatic bud, *LPV* left portal vein, *LVV* left vitelline vein, *M* middle anastomosis, *P* pancreas, *PV* portal vein, *RVV* right vitelline vein, *VP* ventral pancreatic bud

Development of the pancreas occurs concurrently with the formation of the portal venous system, as the ventral pancreatic bud rotates clockwise (viewed from cephalad perspective) around the duodenum to meet its dorsal counterpart, with which it then fuses together (Fig. 4). This normal development places the PV posterior to both the pancreas and the duodenum. In PPPV, by contrast, the dorsal pancreatic bud lies caudal, not cephalad, to the middle anastomosis and dorsal, not ventral, to the left vitelline vein. During the normal clockwise (viewed from above or cephalad) rotation of the ventral bud of the pancreas, the PV will come to lie anterior to the pancreas and posterior to the duodenum. In most of the cases the PV courses in an L-shape or reverse L-shape pattern (Fig. 5) [6]. However, in the more frequent anomaly, a preduodenal PV (where the PV lies anterior to the duodenum), it is usually associated with malrotation of the gut. In this preduodenal anomaly, the dorsal pancreatic bud usually lies in the usual location, cranial to the middle anastomosis. During rotation of the ventral pancreatic bud, the middle anastomosis regresses and the caudal one – which is ventral to the duodenum – becomes the PV (Fig. 6) [16].

## Conclusions

Despite the rarity of PPPV, familiarity of this anomaly is paramount. Knowledge of this anatomic variation and other PV anomalies will help surgeons and interventionalists to avoid catastrophic complications related to inadvertent injury to the PV resulting in massive hemorrhage or ischemic complications to the liver or bowel.
